# Rydberg-Atom-Based Angle-of-Arrival Estimation Method for Ku-Band Microwave Signals

**DOI:** 10.3390/s26165078

**Published:** 2026-08-11

**Authors:** Xingchen Hu, Hao Wu, Yong Gao, Beibei Zhang, Peicheng Liu, Songlin Chen

**Affiliations:** 1 Lab of Quantum Detection & Awareness, Space Engineering University, Beijing 101416, China; hx_chen@hgd.edu.cn (X.H.); liupeicheng@hgd.edu.cn (P.L.); 2 School of Space Command, Space Engineering University, Beijing 101416, China; 3 Earth Observation System and Data Center China National Space Administration, Beijing 100048, China; zbbgaofen@163.com; 4 The Fourth Business Division, Southwest China Institute of Electronic Technology, Chengdu 610045, China; songlinch0061@163.com

**Keywords:** Rydberg-atom, microwave electric-field sensing, EIT-AT splitting, AoA estimation, effective electric-field intensity response function

## Abstract

The determination of the angle of arrival (AoA) of Ku-band microwave signals is of great significance in satellite communications, spectrum monitoring, and national defense security, which has created an urgent demand for high-precision and interference-resistant passive detection techniques. In this study, we propose a Rydberg-atom-based passive measurement method for estimating the AoA of Ku-band incident signals. Based on well-established quantum optical techniques, the proposed method converts the EIT-AT splitting intervals induced by waves or signals incident from different directions in Rydberg atoms within a single vapor cell into the electric-field intensity at the atomic sensor. An electric-field-intensity–angle response model is then established for AoA estimation of a 13.6 GHz wave or signal. Simulation results demonstrate that the proposed method can determine the incident direction within a certain angular range, with an estimation error of less than 3.8°. Without the need for self-calibration and free from interference caused by actively emitted probing electromagnetic waves or reference electric fields, the proposed approach enables AoA determination of incident waves or signals using a single vapor cell with a length of 5 cm.

## 1. Introduction

The angle of arrival of electromagnetic signals plays a critical role in wireless communications [1], passive localization [2], electromagnetic environment sensing [3], and integrated sensing and communication [4]. As the core frequency band for modern satellite communication and radar systems [5], the determination of AoA of Ku-band microwave signals is of great significance in spectrum monitoring and national defense security. Conventional direction-finding methods are predominantly based on active transceiver equipment, which suffers from poor concealment and weak anti-jamming capabilities. Moreover, the detection sensitivity of metallic antennas is fundamentally limited by thermal noise, while their physical size is constrained by the Chu limit [6,7,8]. In addition, antennas exhibit frequency selectivity, and the use of different array configurations in measurements introduces further challenges, such as poor multi-channel consistency and limited bandwidth [9,10]. Meanwhile, in the face of increasingly complex high-frequency electromagnetic environments, research on fine manipulation of electromagnetic fields and metasurface modulation has also received continuous attention [11,12,13,14]. In contrast, Rydberg-atom-based electric field sensors offer higher sensitivity, smaller volume, lighter weight, and broadband signal detection capability [15,16,17,18]. They are capable of passively receiving signals and optically reading out spectral information arising from quantum effects, converting it into instantaneous electric field strength information. This provides a fundamentally new physical paradigm for overcoming the core bottlenecks of traditional direction-finding methods [19,20].

In recent years, the determination of the direction of arrival of electromagnetic waves or signals using Rydberg atoms has attracted considerable attention from researchers worldwide. In 2021, Robinson et al. from the University of Colorado first proposed a Rydberg-atom sensor capable of simultaneously comparing phases to determine the AoA of incident radio-frequency (RF) waves or signals. This technique leveraged the electromagnetically induced transparency (EIT) effect in Rydberg atomic vapor combined with a heterodyne Rydberg-atom mixer, demonstrating the feasibility of atomic-based sub-wavelength phase measurement for determining the direction of incoming waves [21]. Subsequently, Nikunjkumar Prajapti et al. proposed a method for high-resolution imaging of transmitters using Rydberg atoms in a synthetic aperture (SA) configuration, which emulates a two-dimensional array of receivers [22]. Rajavardhan Talashila et al. introduced a sub-wavelength imaging technique for standing-wave fields, utilizing a metal plate to generate a standing-wave pattern without requiring a secondary strong RF phase reference field and independent of incident RF field intensity. This approach achieved high-precision measurements at approximately 5 GHz, yielding a polar-angle resolution of 1.7° over the range of 0° to 60° [23]. In 2026, Oliver et al. employed a dual-ladder Rydberg receiver (DLRR) with RF in-phase detection to achieve direct baseband detection and simultaneous demodulation of the I and Q components of 16QAM, APSK, and QPSK signals. After determining the relative phase of the signals, they successfully estimated the Direction of Arrival(DoA) of the modulated signals [24]. Meanwhile, in 2025, the U.S. Army Research Laboratory proposed an electrically small Rydberg-atom electric field sensor capable of extracting the k-vector in three dimensions for elliptically polarized RF fields, offering a novel approach for determining the propagation direction of electromagnetic waves [25]. In 2026, Yuanbin Chen and his team from Nanyang Technological University, Singapore, proposed a quantum-enhanced angle-resolved polarization-sensitive DoA detection scheme utilizing the intrinsic vector sensitivity of a single Rydberg atomic vapor cell. Their work demonstrated that, with appropriately chosen polarization and magnetic field geometries, a single vapor cell can achieve angular resolution below 0.1° under moderate RF driving strengths [26].

Domestically, several research groups are also dedicated to improving the accuracy and dimensionality of phase-based direction finding. A research team from Shanxi University theoretically proposed and experimentally demonstrated a Ku-band three-dimensional localization system using an L-shaped array of Rydberg-atom receivers [27]. A research group at the National University of Defense Technology constructed a binary array of Rydberg-atom field probes (RFPs) based on Rydberg-atom heterodyne mixers and applied digital beamforming (DBF) techniques, achieving an AoA measurement variance of less than 1.5° [28]. Researchers at Northwestern Polytechnical University reported the first simultaneous measurement of Doppler frequency shift (DFS) and AoA within a single Rydberg-atom system, achieving an AoA measurement error of less than 0.5° over an angular range from −35° to 30° [29].

To fully exploit the physical advantages of Rydberg atoms in electric field sensing and to achieve a more compact and conceptually simpler electromagnetic wave AoA detection scheme, this study proposes and validates a passive method for estimating the AoA of Ku-band incident signals using Rydberg atoms. We establish a single-channel effective electric field strength–angle response model under a fixed propagation distance, fit the effective electric field intensity response function, and perform angle inversion validation and feasibility analysis. Furthermore, electromagnetic simulations are conducted to normalize and analyze the angular response under multiple propagation distances, along with error analysis. Simulation results demonstrate that the proposed method is capable of detecting the AoA of 13.6 GHz electromagnetic waves over the range of 15° to 60°, with an estimation error of less than 3.8°. Compared with phase-difference-based approaches and wave-vector measurement methods, our scheme offers a conceptually simpler single-vapor-cell direction-finding architecture, while also achieving lower measurement errors than conventional antenna-based methods, thereby providing a new perspective for Rydberg-atom-based AoA determination.

## 2. Theoretical Analysis and Modeling

### 2.1. Atomic Coherence Effects and Electric Field Sensing

The microwave electric-field sensing mechanism employed in this work is based on the EIT effect in Rydberg atoms and the microwave-field-induced Autler–Townes (A-T) splitting. For a typical four-level, two-photon excitation system of cesium atoms, the probe and coupling lasers jointly act on the atomic energy-level structure, coherently superposing the ground state and the Rydberg state to form a dark state. In this dark state, the absorption of the probe light by the atoms is significantly suppressed, thereby producing a narrow-linewidth transparency window in the absorption spectrum, which is the EIT phenomenon. When an external microwave field resonantly couples two adjacent Rydberg states, the EIT spectrum splits into an Autler–Townes doublet structure. The frequency interval between the two split peaks satisfies the following relation $f_{A-T}$ with the microwave-field-induced Rabi frequency $\Omega_{MW}$:

(1) $$f_{A-T}=D\frac{\Omega_{MW}}{2\pi },$$

where the probe laser is frequency-locked and the coupling laser is scanned, resulting in a Doppler mismatch factor D=1 in this relation. Under the dipole approximation, the interaction between the microwave electric field and the Rydberg transition yields the Rabi frequency:

(2) $$\Omega_{MW}={℘}_{MW}\cdot \left|E_{MW}\right|/\hbar ,$$

where ${℘}_{MW}$ denotes the transition dipole moment between the two Rydberg levels, which can be obtained using atomic structure calculation tools such as the Alkali Rydberg Calculator (ARC); $\left|E_{MW}\right|$ is the microwave electric field strength; and ℏ is the reduced Planck constant. Combining (1) and (2), it follows that the A-T splitting interval is proportional to the electric field strength, which can be expressed as:

(3) $$\left|E_{MW}\right|=\frac{\hbar }{{℘}_{MW}}\Omega_{MW}=\frac{\hbar }{{℘}_{MW}}2\pi f_{A-T}.$$

Therefore, by exploiting the atomic coherence effects arising from the interaction between cesium atoms and the two laser fields as well as the microwave field, the measurement of the microwave field amplitude can be converted into an optical frequency measurement, i.e., the frequency difference of the A-T splitting in the EIT signal.

### 2.2. Field–Angle Response Direction-Finding Model

In optical frequency measurements, the frequency difference of the A-T splitting originates from the response of the atoms within the overlapping region of the probe and coupling beams to the applied microwave field. To simplify the analysis, the effective measurement region is approximated as a cylindrical volume formed by the spatial overlap of the probe and coupling beams within their full width at half maximum (FWHM) dimensions, denoted by V:

(4) $$V=\pi {\left(\frac{d_{FWHM}}{2}\right)}^{2}L,$$

where $d_{FWHM}$ is the transverse FWHM beam radius and L is the length between the inner walls of the vapor cell. Since the actual atomic response is affected by the spatially non-uniform weighting resulting from the Gaussian intensity distributions of the probe and coupling beams, in order to verify the theoretical and electromagnetic-simulation feasibility of the single-vapor-cell passive AoA scheme, this paper adopts a first-order approximation of uniform volume averaging, which reflects the spatially averaged result of the electric-field amplitude over the effective volume. In this paper, the effective electric field strength is defined as:

(5) $$E\left(\theta ,R\right)=\frac{1}{V}\int_{V}\left|E\left(r;\theta ,R\right)\right|dV,$$

where θ denotes the incident angle of the microwave field, R is the propagation distance from the source to the center of the atomic antenna, and $\left|E\left(r;\theta ,R\right)\right|$ is the local electric field amplitude at position r within the effective volume.

Accordingly, considering both the distance-dependent attenuation of the incident microwave signal during free-space propagation and the structural directional response of the Rydberg-atom vapor cell to different incidence directions, the effective microwave electric field strength is expressed as:

(6) $$E\left(\theta ,R\right)=T\left(\theta \right)\cdot A\left(R\right),$$

where $T\left(\theta \right)$ denotes the directional response function of the Rydberg-atom vapor cell, describing the normalized field-strength response corresponding to different incidence angles at a fixed propagation distance; and $A\left(R\right)$ is the incident field amplitude factor determined by the propagation distance R, primarily reflecting free-space path loss and environmental propagation effects.

Under the condition that the microwave source radiates stably and the propagation distance R is known or remains constant, the single-channel direction-finding problem can be reformulated as an angle inversion problem based on the electric-field-strength response function. Furthermore, within a given operational angular range $\left[a,b\right]$, if the fitted function relating the electric field strength to the incident angle $E_{fit}\left(\theta ,R\right)$ satisfies:

(7) $$\frac{\partial E_{fit}\left(\theta ,R\right)}{\partial \theta }\neq 0,\forall \theta \in \left[a,b\right],$$

and its inverse function exists, the microwave incidence angle can be expressed as:

(8) $${\hat{\theta }}_{est}=E_{fit}^{-1}\left[E_{fit}\left(\theta ,R\right)\right],$$

Thus, the fitting error model for the field-strength–incident-angle relationship is established as:

(9) $$\delta_{\theta_{est}}\left(\theta \right)=\left|{\hat{\theta }}_{est}-\theta \right|=\left|E_{fit}^{-1}\left[E_{fit}\left(\theta \right)\right]\right|,$$

In practical measurements, combining (3) and (6), the A-T splitting response model of the Rydberg-atom sensor can be written as:

(10) $$f_{A-T}=\frac{{℘}_{MW}}{2\pi \hbar }E_{fit}\left(\theta ,R\right),$$

Under the condition that the microwave source radiates stably and the propagation distance R is known or remains constant, the A-T splitting frequency interval varies only with the incident angle. In this case, letting $\beta ={℘}_{MW}/2\pi \hbar$, the measured microwave incidence angle can be computed as:

(11) $${\hat{\theta }}_{meas}=E_{fit}^{-1}\left(\frac{{\hat{f}}_{A-T}}{\beta }\right),$$

where ${\hat{f}}_{A-T}$ is the optically measured A-T splitting value. Owing to the influence of atomic background noise, optical components, and environmental noise, the sources of measurement error are complex and multifaceted. The error model can be evaluated using the following expression:

(12) $$\delta_{\theta_{meas}}\left(\theta \right)=\left|{\hat{\theta }}_{meas}-\theta \right|=\left|E_{fit}^{-1}\left[E_{fit}\left(\theta \right)\right]\right|.$$

## 3. Full-Wave Simulation and Validation

### 3.1. Electromagnetic Simulation Scenario Setup

**Figure 1:**
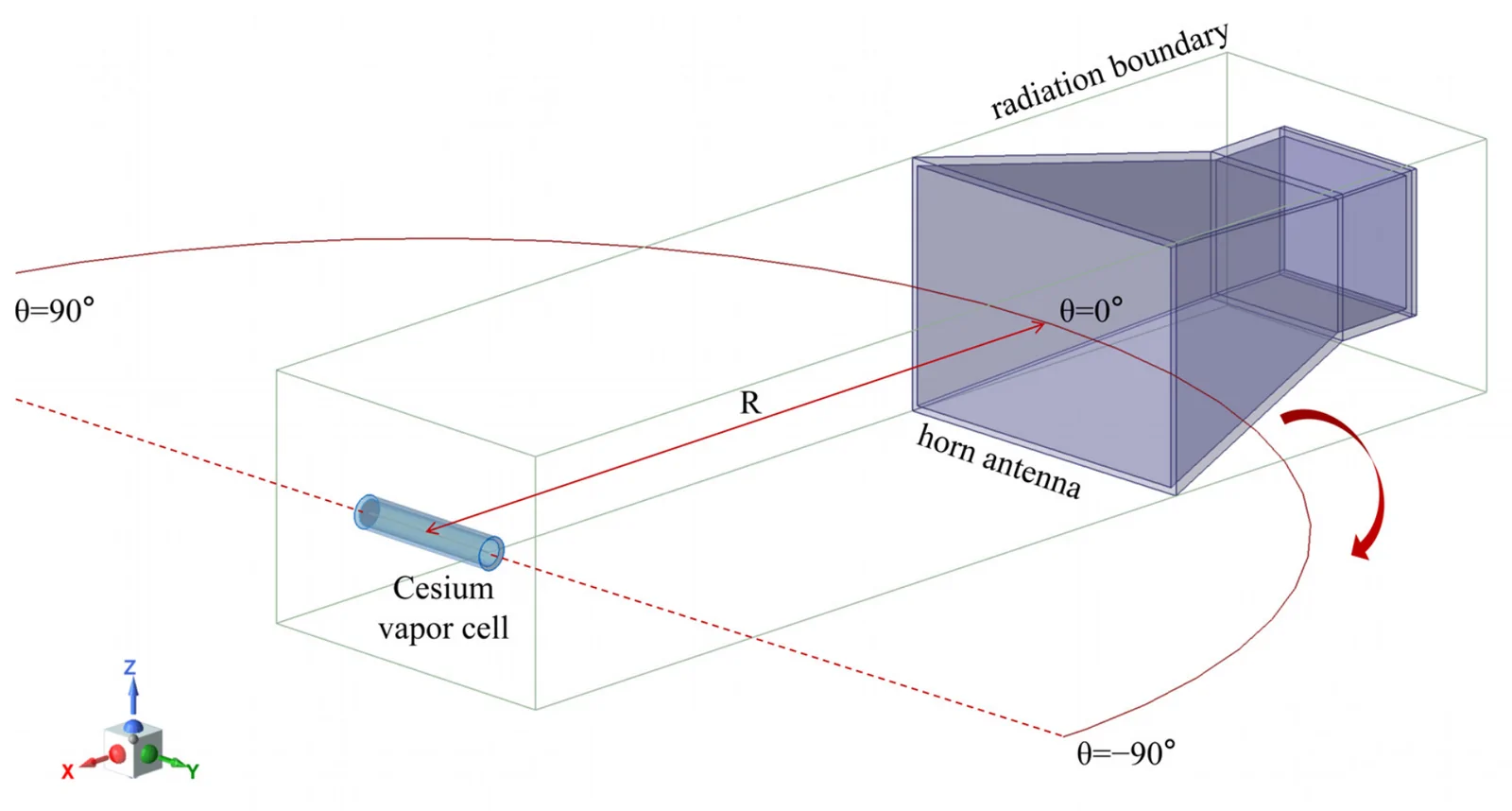
Schematic of the three-dimensional model structure.

To study the directional response characteristics of the Rydberg-atom sensor to microwave incident field signals in the x-y plane, we employ Ansys HFSS (High Frequency Structure Simulator) to model the microwave electric field SIG and the atomic vapor cell, as shown in Figure 1.

The atomic vapor cell is a cylindrical glass cell filled with cesium alkali metal vapor, with a diameter of 10 mm, a length of 50 mm, and a wall thickness of 1 mm. A small blow-molded gas port is present on the sidewall surface. The central axis of the cell is designated as the reference line for effective electric field observation. To more realistically simulate the microwave scenario, the 852 nm probe laser and 509 nm coupling laser for preparing the Rydberg states of cesium atoms are placed in the same horizontal plane of the vapor cell, and the FWHM is set as the diameter of the effective measurement region for the electric field strength. The horn antenna, with its aperture facing the horizontally placed cesium vapor cell, operates at a frequency of 13.6 GHz with an input power of 1 W, serving as the SIG source for the incident microwave field. The distance from the antenna aperture to the center of the atomic vapor cell is defined as the signal propagation distance R, which can be approximated as satisfying the far-field condition. The outer boundary of the simulation domain is set as a Radiation boundary condition, with the radiation boundary placed at a distance of 11.02 mm from the outer surface of the main structure, which is approximately half a wavelength in vacuum, so as to reduce the influence of boundary reflections on the near-field distribution. To ensure that the near-field interaction between the metallic horn antenna and the dielectric glass wall of the vapor cell is accurately resolved, HFSS adaptive meshing and iterative solver strategies are adopted. Owing to the cylindrical symmetry of the vapor cell, we rotate the antenna in the x–y plane to simulate different incidence directions θ ranging from −90° to 90° with a step size of 1°. When the horn antenna is rotated to the center of the sidewall of the cell, the incident angle is defined as θ=0. The simulation region is configured as a free-space solution domain with radiation boundaries to emulate an open environment and minimize reflection effects. To ensure computational accuracy, the simulation domain is set to be significantly larger than the operating wavelength. The distribution of the effective electric field E is obtained from HFSS and further analyzed.

### 3.2. Feasibility Analysis of the Direction-Finding Method Based on the Field-Strength–Incident-Angle Relationship

**Figure 2:**
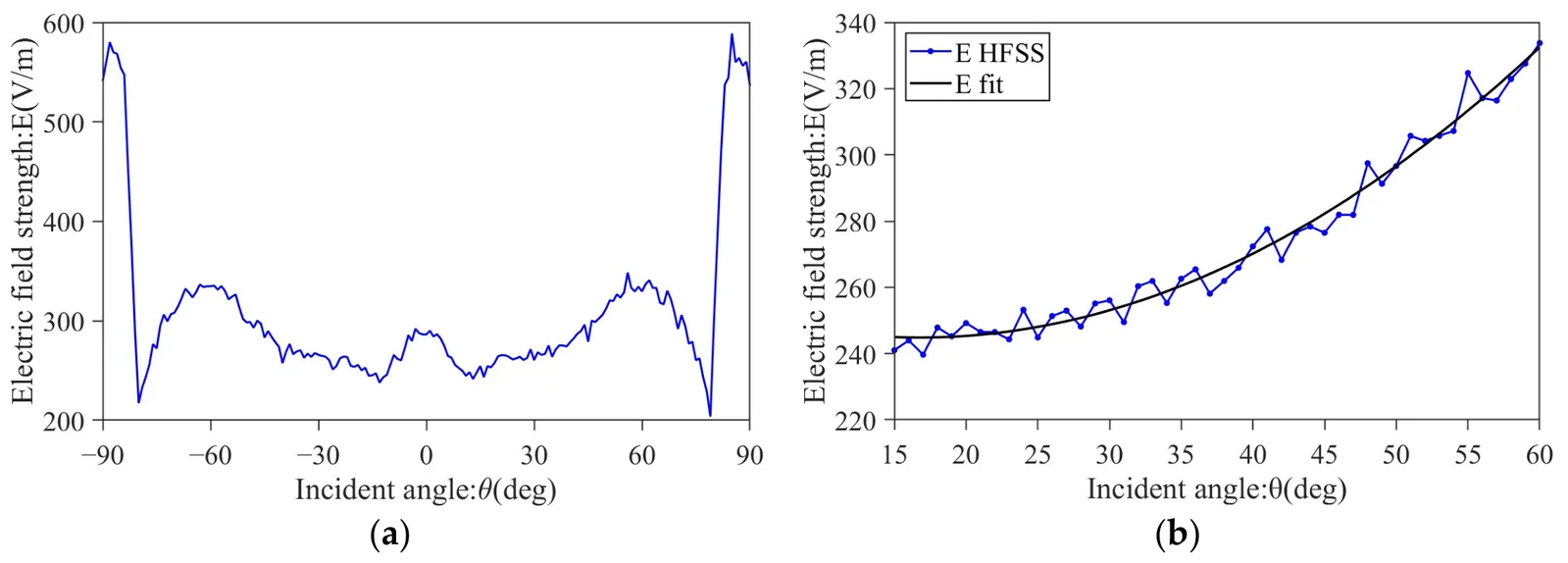
HFSS-simulated field-strength incident-angle data relationship. (**a**) Simulated field-strength angle mapping over the full 180° range; (**b**) Function fitting of field-strength angle relationship over the 15–60° interval.

Based on the established HFSS electromagnetic simulation model, we implement automated angular scanning of the incident microwave field using a Python3.13 co-simulation approach. At a fixed propagation distance R=25 cm, the electric field strength within the effective interaction volume between the microwave field and the Rydberg atoms is calculated and acquired by varying the signal incidence angle θ. A total of N=181 angular sampling points are obtained from HFSS, and the simulated multi-distance directional response results are presented in Figure 2a. Based on a comprehensive consideration of monotonicity characteristics and the angular measurement range, a specific angular interval θ∈ [15°, 60°] is selected, and the variation curve of the effective electric field strength with respect to the incident angle $E\left(\theta ,R=25\;\mathrm{cm}\right)$ is obtained, as shown by the blue polyline in Figure 2b. It can be observed that the electric field strength exhibits an approximately linear relationship with the incident angle overall; however, fluctuations exist within adjacent small angular ranges, preventing a strict one-to-one correspondence between the effective electric field strength and the angle. Therefore, a fitting procedure is applied. Considering the large fitting deviation of low-order fitting and the complexity penalty along with strict monotonicity screening for high-order fitting, a second-order polynomial fitting $E_{fit}\left(\theta ,R=25\;\mathrm{cm}\right)$, which yields the best fitting performance, is ultimately selected, as shown by the black curve in Figure 2b.

**Figure 3:**
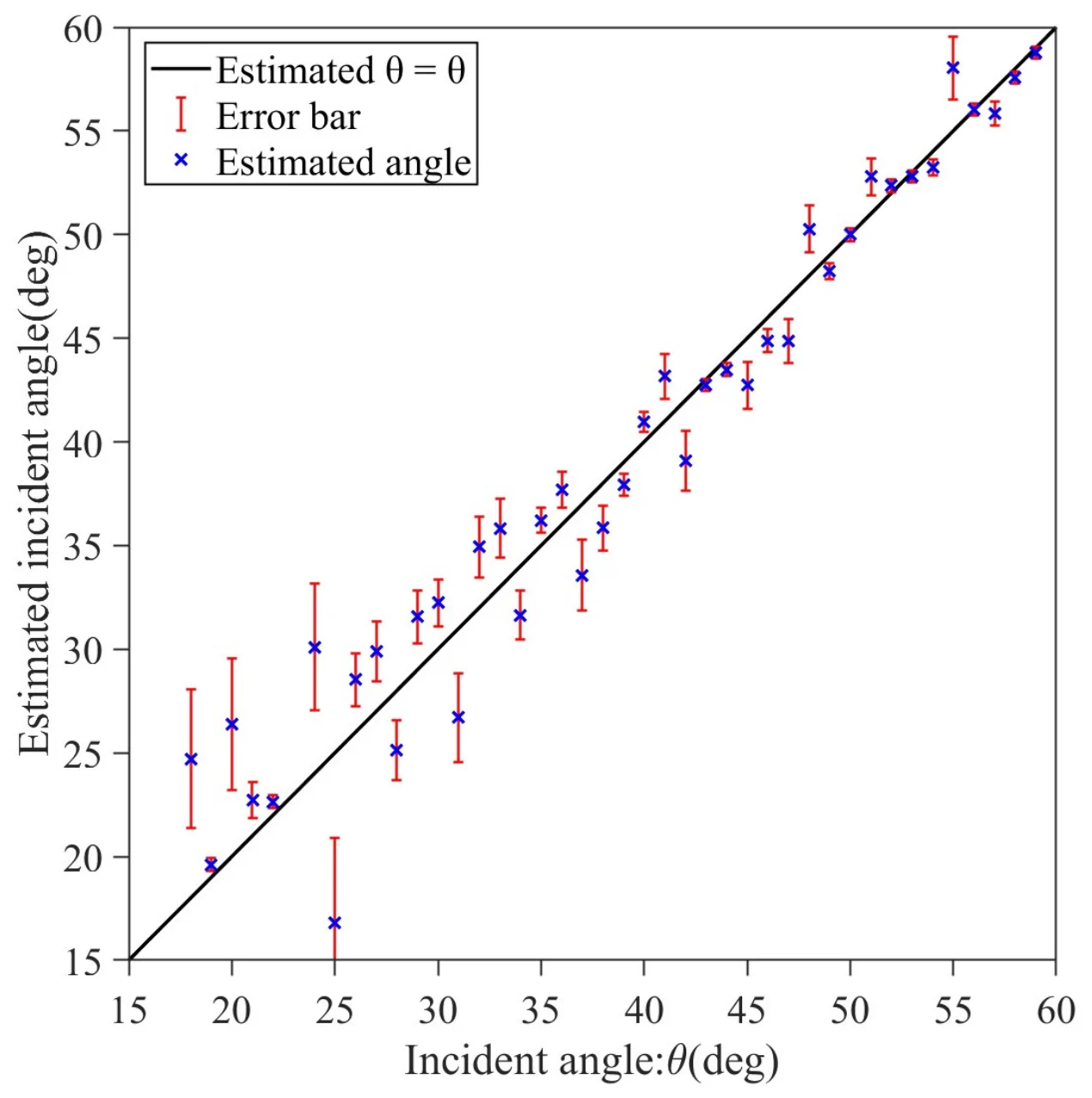
Direction-finding data based on the field-strength–incident-angle relationship.

To further validate the feasibility of the AoA method based on the effective electric-field-strength response fitting function, we perform angle inversion using the second-order fitting function $E_{fit}\left(\theta ,R=25\;\mathrm{cm}\right)$ and compare the estimated incidence angles ${\hat{\theta }}_{est}$ with the true angles θ. Figure 3 presents the angle estimates computed from the HFSS results using the second-order fitting function over the selected angular range θ∈ [15°, 60°]. The solid line in the figure denotes the one-to-one correspondence between the true incidence angle and the simulated estimated values. It can be observed that the AoA estimates generally follow this solid line, though they do not lie exactly on it, which may be attributed to estimation bias introduced by the fitting function. Meanwhile, the absolute error analysis of the fitting-function-based direction-finding method, as indicated by the red error bars in Figure 3, shows that the angle estimation errors fall within the range of 5°, demonstrating a certain degree of directional specificity. Therefore, within the selected monotonic interval, the angle inversion method based on the second-order fitting model exhibits satisfactory feasibility.

**Figure 4:**
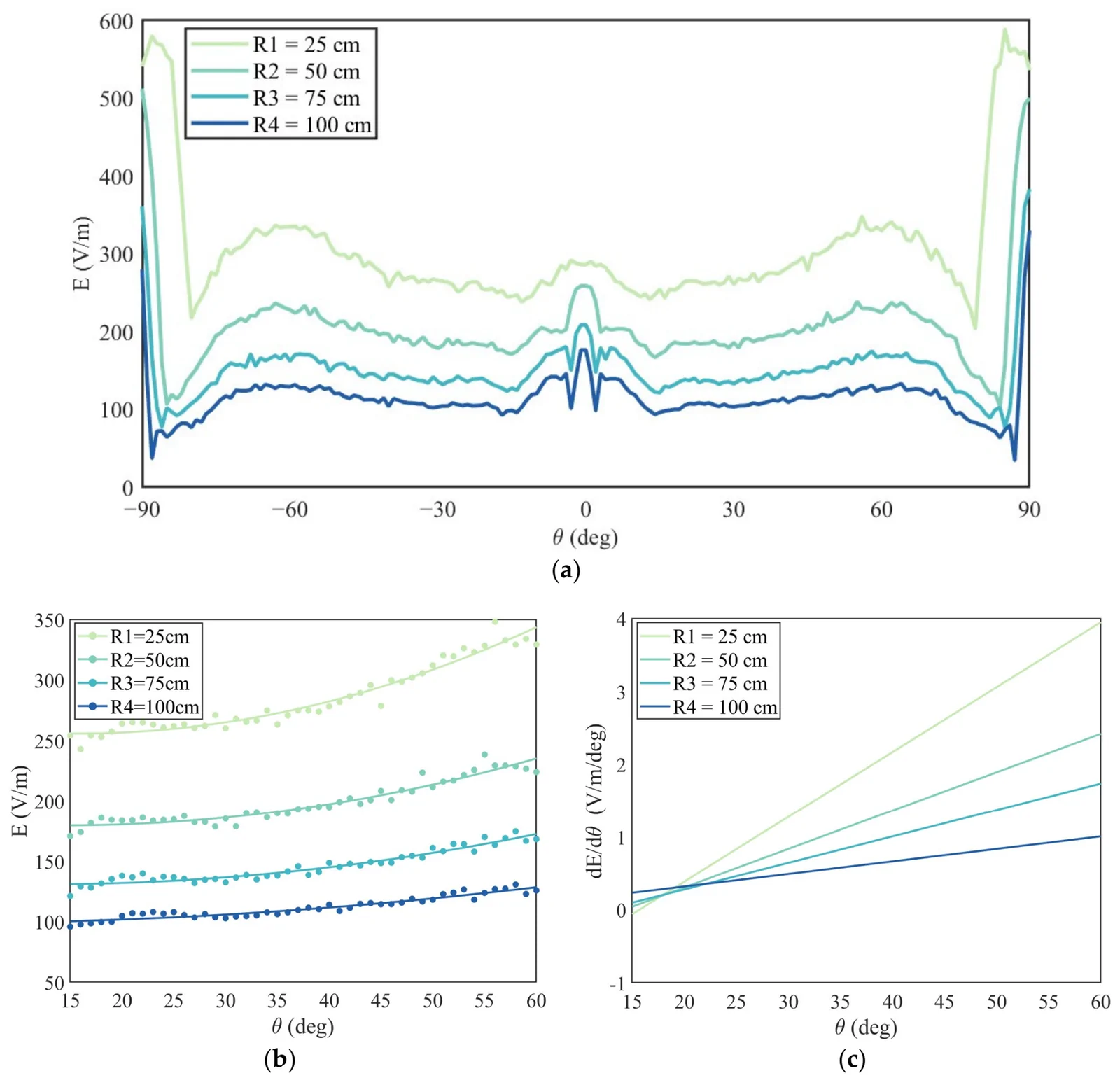
Directional response curves for multiple propagation distances. (**a**) Field-strength response curves under different propagation distances; (**b**) Second-order fitting functions over the 15–60° scan range; (**c**) Gradient values of the second-order fitted directional responses.

To further investigate the influence of propagation distance on the directional response characteristics, we select four different microwave signal propagation distances $R_{i}\in \left\{25,50,75,100\right\}\mathrm{cm},i=1,2,3,4$ and perform automated angular scanning simulations at each distance with a step size of 1° over the range θ∈ [−90°, 90°]. A total of 4 $N_{i}=181$ angular sampling points are obtained from HFSS, and the simulated multi-distance directional response results are presented in Figure 4a. From the simulation results, it can be observed that the directional response curves under different propagation distances maintain consistent overall trends, with each curve exhibiting similar angular variation characteristics. As the propagation distance increases, the overall amplitude of the curves decreases, while the curve shapes remain essentially unchanged, primarily manifesting as a global scaling in amplitude. This demonstrates that the vapor cell structure possesses directional directivity. However, it can also be observed that the electric-field response exhibits a symmetric distribution between −90° and 0° and between 0° and 90°, which leads to ambiguity in distinguishing positive and negative angles over the 180° range. If the angular measurement range is not restricted, a single vapor cell with an axisymmetric structure cannot meet the requirement for large-range angle estimation, and it is necessary to further investigate the feasibility of dual-cell, multi-cell cooperative measurement, or array-based layout approaches. Furthermore, owing to small-scale fluctuations in the curves, which render them non-strictly monotonic and prevent a strict one-to-one correspondence between effective electric field strength and angle, fitting is performed on the simulated values under the four propagation distances. Considering the complexity penalty of high-order fitting and strict monotonicity screening, a second-order polynomial fitting, which yields the best fitting performance, is ultimately adopted, as shown in Figure 4b. With increasing propagation distance, the microwave power attenuates, resulting in a gradual decrease in the slope of the directional response curve within this interval, leading to reduced angular resolution capability. The gradient relationships of the fitted curves are established, as shown in Figure 4c.

**Figure 5:**
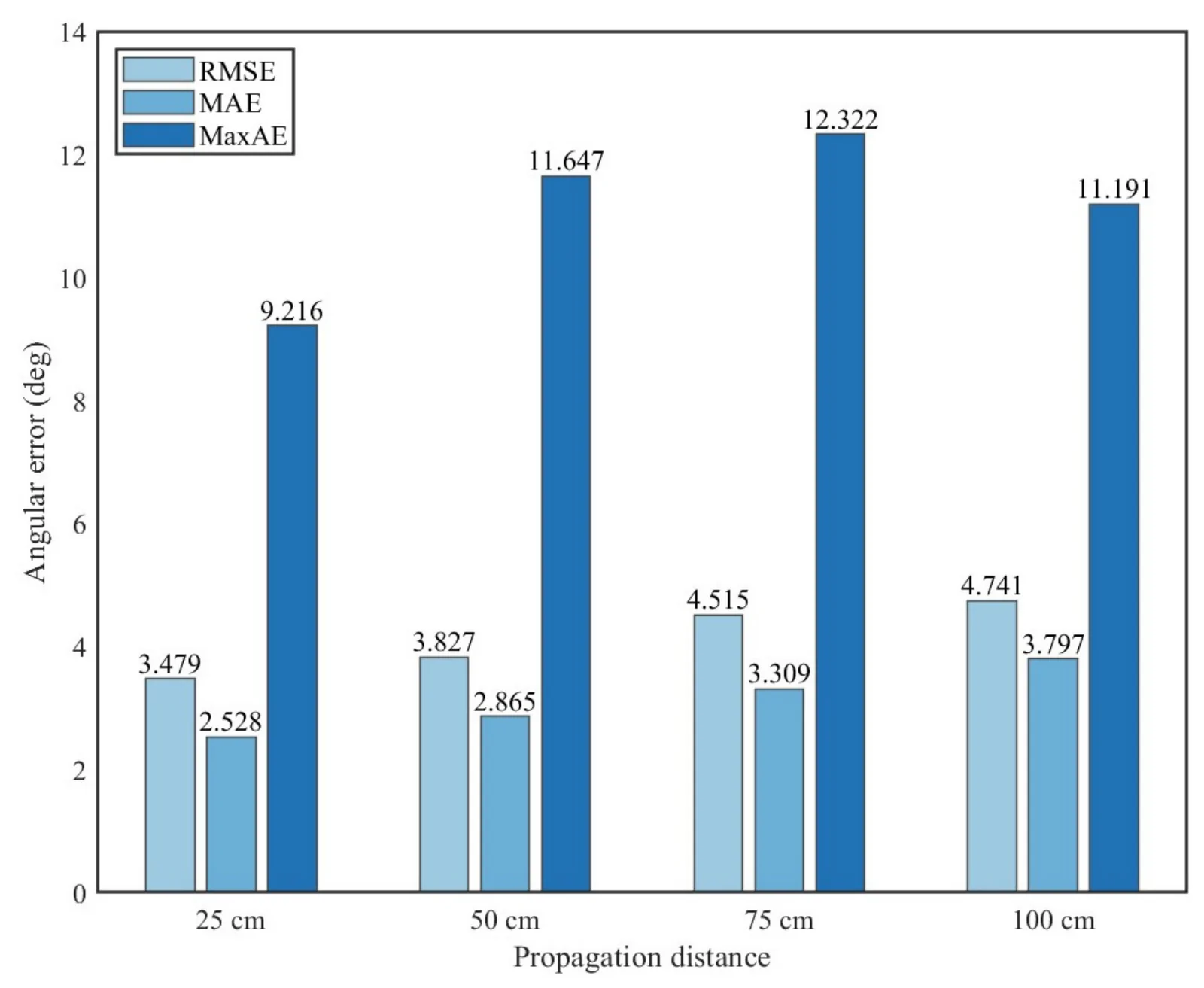
Histogram of direction-finding metrics.

To quantitatively evaluate the direction-finding accuracy, we further employ the root mean square error (RMSE), mean absolute error (MAE), and maximum absolute error (MaxAE) as evaluation metrics. Figure 5 presents the statistical results of these error metrics for the four propagation distances. It can be observed that the RMSE effectively reflects the overall direction-finding accuracy, with error levels within ±5°. As the propagation distance increases, both RMSE and MAE exhibit an increasing trend. This observation is consistent with the preceding analysis of the gradient of the electric-field-strength incident-angle response function, indicating that the sensitivity of the atomic sensor to changes in the microwave incidence angle decreases, thereby compromising angular resolution capability. Consequently, under the same amplitude measurement error conditions, the angle inversion error is amplified with increasing propagation distance.

It is also noteworthy that, in this experiment, the field-strength ranges of the field-strength angle fitting functions corresponding to the four selected propagation distances do not overlap within the investigated range θ∈ [15°, 60°]. Therefore, even when the specific propagation distance among these four is unknown, the AoA of incoming waves within this angular range can still be determined. However, it should be acknowledged that the proposed method cannot eliminate the angle ambiguity arising from arbitrary propagation distances.

## 4. Conclusions

In this study, based on well-established quantum optical techniques, we proposed a passive angle-of-arrival measurement method for Ku-band incident signals using Rydberg atoms. The EIT-AT splitting intervals induced by waves or signals incident from different directions in Rydberg atoms within a single vapor cell were converted into the electric-field intensities at the atomic sensor. A directional response model between the electric field in the atom–field interaction region and the propagation direction of the incident electromagnetic wave was then established. Angle inversion was achieved by fitting the response function, and the estimation performance was evaluated using error metrics represented by the root mean square error. The results show that the proposed amplitude-based angle-of-arrival estimation method can effectively estimate the incident direction. In addition, HFSS simulations at multiple propagation distances verified the stability of the directional response.

Compared with phase-based direction-finding methods using Rydberg atoms, the proposed method has a simpler operating principle, avoids interference from reference electromagnetic fields, and relies only on a single atomic vapor cell, making it more suitable for highly integrated receiver systems. Admittedly, there are also some engineering challenges in the implementation of this method. First, in the process of determining the propagation distance of the electromagnetic wave and, at this distance, calibrating the fitted relationship between the field strength and the incident angle over a certain angular range by using a fixed field source and rotating the atomic vapor cell to change the precise incident angle, and reading out the A-T splitting information from the photodetector to determine the AoA, various sources of error, such as ranging errors, calibration errors, and spectral readout errors, will arise at different levels. These need to be optimized to achieve more accurate angle-of-arrival information. Second, the temperature of the vapor cell affects the density distribution of alkali metal atoms; therefore, the experimental environment needs to be strictly temperature-controlled at room temperature to stabilize the atomic distribution. The effects of clutter and standing waves caused by reflections from metallic components cannot be ignored either. It is necessary to properly place absorbing materials, and even use custom-designed non-metallic structural support materials in the integrated optical path, to purify the experimental environment. In addition, the results presented in this paper are mainly based on simulation analyses under idealized readout conditions, and actual experimental factors may further affect the spectral A-T splitting information and thus the angle-estimation accuracy. In future work, we will refine the model by incorporating experimental conditions, aiming to enhance the direction-finding performance of the system while maintaining manageable system complexity.

## Data Availability

Data is contained within the article. The original contributions presented in this study are included in the article. Further inquiries can be directed to the corresponding author.
